# Sea ice drift data for Fram Strait derived from a feature-tracking algorithm applied on Sentinel-1 SAR imagery

**DOI:** 10.1016/j.dib.2018.04.034

**Published:** 2018-04-19

**Authors:** Stefan Muckenhuber, Stein Sandven

**Affiliations:** Nansen Environmental and Remote Sensing Center (NERSC), Thormøhlensgate 47, 5006 Bergen, Norway

## Abstract

1541 Sentinel-1 SAR images, acquired over Fram Strait between 2014 and 2016, were considered for sea ice drift retrieval using an open-source feature tracking algorithm. Based on this SAR image data set, 2026 and 3120 image pairs in HH and HV polarisation were used to calculate monthly mean sea ice velocities at 79 N.

**Specifications table**

**Table:** 

Subject area	Physics
More specific subject area	Remote sensing of sea ice
Type of data	DAT files, python scripts, plots
How data was acquired	Computer server
Data format	Raw, filtered and analyzed
Experimental factors	Preprocessing of SAR data
Experimental features	A feature tracking algorithm is used to derive sea ice drift from Sentinel-1 SAR images
Data source location	Fram Strait
Data accessibility	With this article
Related research article	Muckenhuber S., Korosov A.A., Sandven S. (2016): Open-source feature-tracking algorithm for sea ice drift retrieval from Sentinel-1 SAR imagery, The Cryosphere, 10, 913–925, doi:10.5194/tc-10-913-2016, 2016.

**Value of the data**

● The presented dataset shall serve for a better understanding of the sea ice export through Fram Strait.
● The dataset allows to study the capabilities of a computationally efficient feature-tracking algorithm to derive sea ice drift from large Sentinel-1 SAR image data sets.
● These efforts shall contribute to the preparation of an operational high-resolution sea ice drift product based on Sentinel-1 SAR imagery.

## 1 Data

Fram Strait represents a major gateway between the Arctic Ocean and the Atlantic Ocean, both in terms of oceanic heat transport into the Arctic and sea ice export into lower latitudes. About 10% of the Arctic sea ice cover is exported through Fram Strait every year [6].

A total of 1541 Sentinel-1 SAR images, acquired over Fram Strait in the time period October 2014–February 2016, have been considered for automatic sea ice drift retrieval. Image pairs were chosen according to overlapping area and a time difference of less than 72 h.

A feature tracking algorithm from Muckenhuber et al. [3] was applied on both HH and HV polarisation to include the information of both channels and to investigate the polarisation dependence on the feature tracking performance. Image pairs that provided less than 200 drift vectors were rejected. This resulted in a total of 2026 and 3120 image pairs for HH and HV polarisation respectively.

**Fig. 1:**
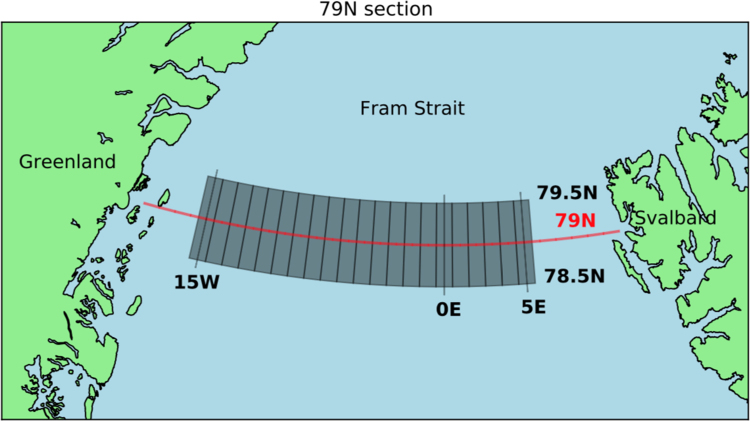
Map of Fram Strait displaying considered area (grey boxes) for SAR sea ice drift algorithm and 79 N Section (red).

To overcome the different vector distributions and time gaps of the image pairs, the monthly mean was calculated as the weighted mean over all vectors found during 1 month in 1 grid cell (shown as grey boxes in Fig. 1). Monthly data points that included less than 20 drift vectors were rejected. The weighting function considered the time interval between the two image acquisitions, i.e. time difference between first and second image, divided by the maximum time difference of 72 h.

## 2 Experimental design, materials, and methods

The Sentinel-1 mission consists of two identical satellites (Sentinel-1A launched in 2014, Sentinel-1B launched in 2016), each carrying a C-band Synthetic Aperture Radar (SAR) that measures radar backscatter at a centre frequency of 5.405 GHz. The revisit time over Fram Strait is less than one day and all images are distributed via the Copernicus services with open and free access.

The used algorithm is designed for Sentinel-1 images taken in’Extra Wide Swath Mode Ground Range Detected with Medium Resolution’ mode. This is the main acquisition mode of Sentinel-1 for sea ice covered areas. The covered area per image is 400 km×400 km with a pixel spacing of 40 m×40 m, a resolution of 93 m range×87 m azimuth and less than 10 m residual planimetric distortions. Both HH (horizontal transmit, horizontal receive) and HV (horizontal transmit, vertical receive) polarisation are provided in the considered mode.

Sea ice drift retrieval algorithms using high resolution SAR imagery are based either on pattern matching or feature tracking, with the latter being considered computationally more efficient. Considering a large dataset including several thousand image pairs, we have decided to use an open-source feature-tracking algorithm for Sentinel-1 SAR imagery, published by Muckenhuber et al. [3]. The applied algorithm was trained for our area of interest and has been shown to be computationally very efficient with a processing time of approximately one minute per image pair.

The SAR sea ice drift algorithm introduced by Muckenhuber et al. [3] is based on the feature-tracking algorithm ORB (Oriented FAST and Rotated BRIEF) by Rublee et al. [5] and includes three main steps:

– First, keypoints are detected in both images on several resolution-pyramid levels using the FAST-9 keypoint detector from Rosten and Drummond [4]. A pixel is recognised as keypoint, if nine contiguous pixels in the surrounding circle, with perimeter 16 pixels, are brighter or darker than the centre pixel plus threshold.
– Second, a 34×34 pixels area around each keypoint is considered for feature description. A modified version of the binary descriptor BRIEF [1] returns a feature vector with 256 binary values for each keypoint.
– Third, all feature vectors of the first image are compared to all feature vectors of the second image (Brute Force Matching) and the best two results are returned. The better match is accepted, if the ratio test from Lowe [2] returns a value below 0.75.

The resulting feature-tracking vectors show a high degree of independence in terms of position, lengths and direction, which can allow resolving displacement gradients on a very high resolution. On the other hand, the distribution of the vector field is determined by the feature recognition performance of the image and cannot be chosen. This makes the inter-comparison with other drift fields more challenging.

**Fig. 2:**
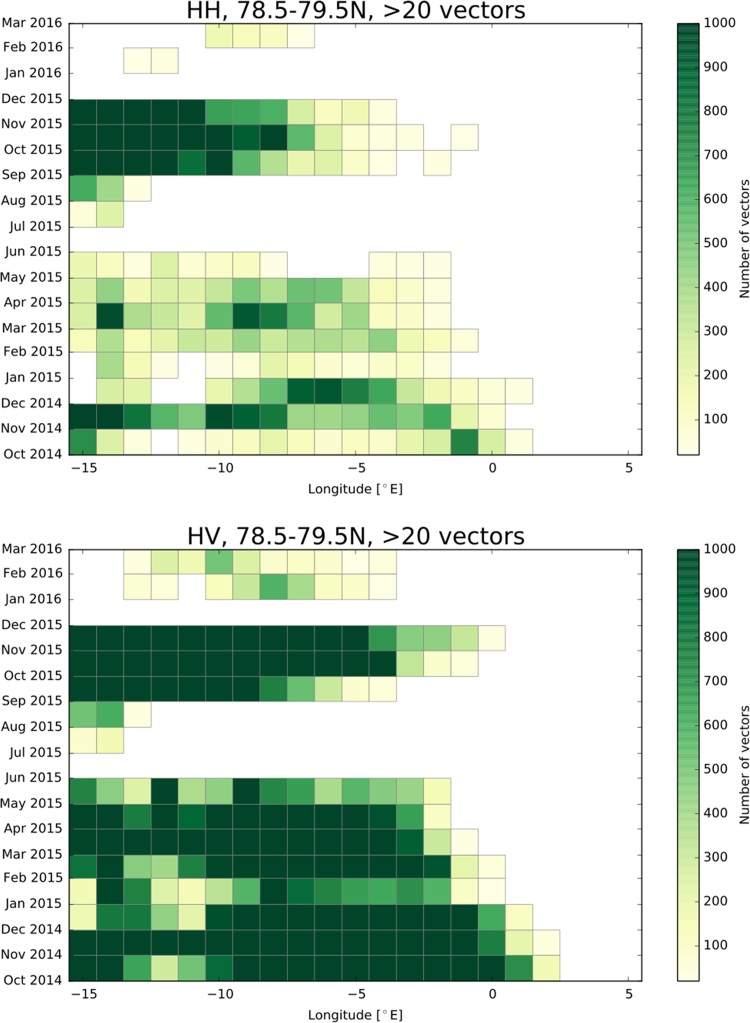
Number of detected drift vectors derived from HH and HV polarisation.

**Fig. 3:**
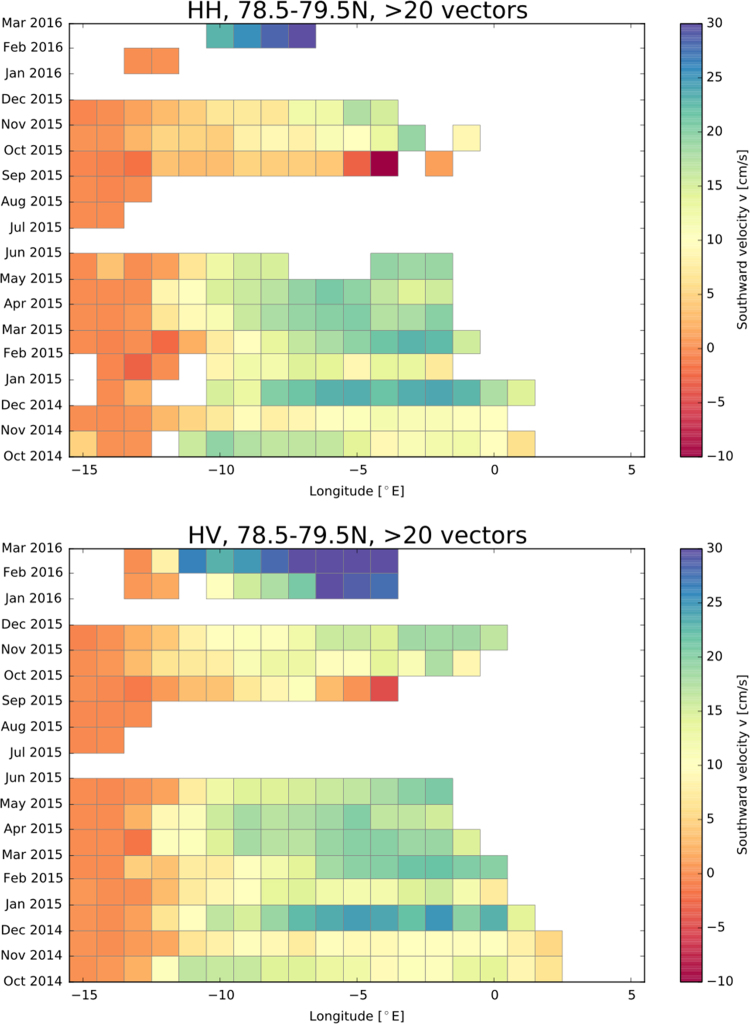
Southward velocity component in cm/s derived from HH and HV polarisation.

**Fig. 4:**
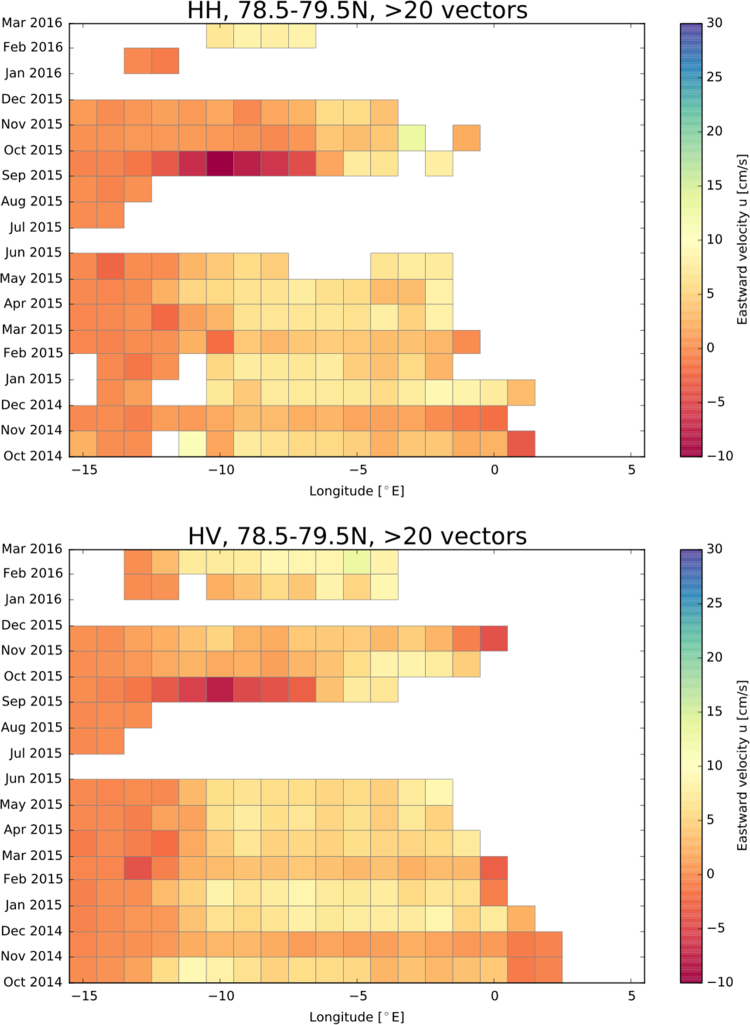
Eastward velocity component in cm/s derived from HH and HV polarisation.

Fig. 2 shows the number of detected drift vectors derived from HH and HV polarisation and Fig. 3, Fig. 4 depict the southward and eastward velocity component in cm/s derived from HH and HV polarisation.
