# Supplementary material for: Sea ice drift data for Fram Strait derived from a feature-tracking algorithm applied on Sentinel-1 SAR imagery
Source: Data Brief. 2018 Apr 19;18:1410–5. doi: 10.1016/j.dib.2018.04.034 (PMC5999601; doi:10.1016/j.dib.2018.04.034)
Supplement: Supplementary file 2 — Supplementary material [file mmc2.zip › fs_s1a_plots/fs_s1a_map.pdf]

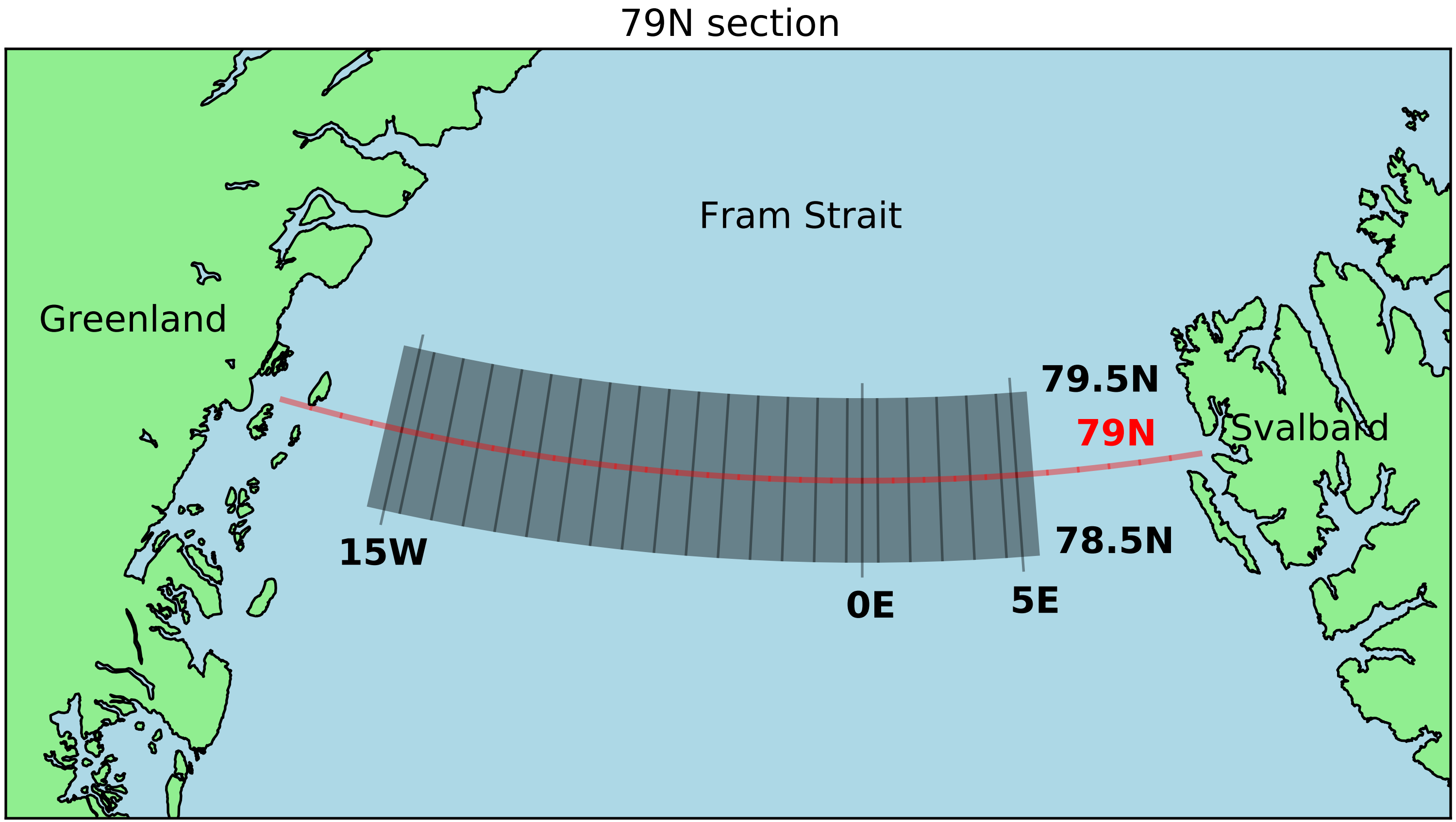
79N section
Fram Strait
Greenland
Svalbard
79.5N
79N
78.5N
15W
0E
5E
