# Supplementary material for: Sea ice drift data for Fram Strait derived from a feature-tracking algorithm applied on Sentinel-1 SAR imagery
Source: Data Brief. 2018 Apr 19;18:1410–5. doi: 10.1016/j.dib.2018.04.034 (PMC5999601; doi:10.1016/j.dib.2018.04.034)
Supplement: Supplementary file 2 — Supplementary material [file mmc2.zip › fs_s1a_plots/HV_n.pdf]

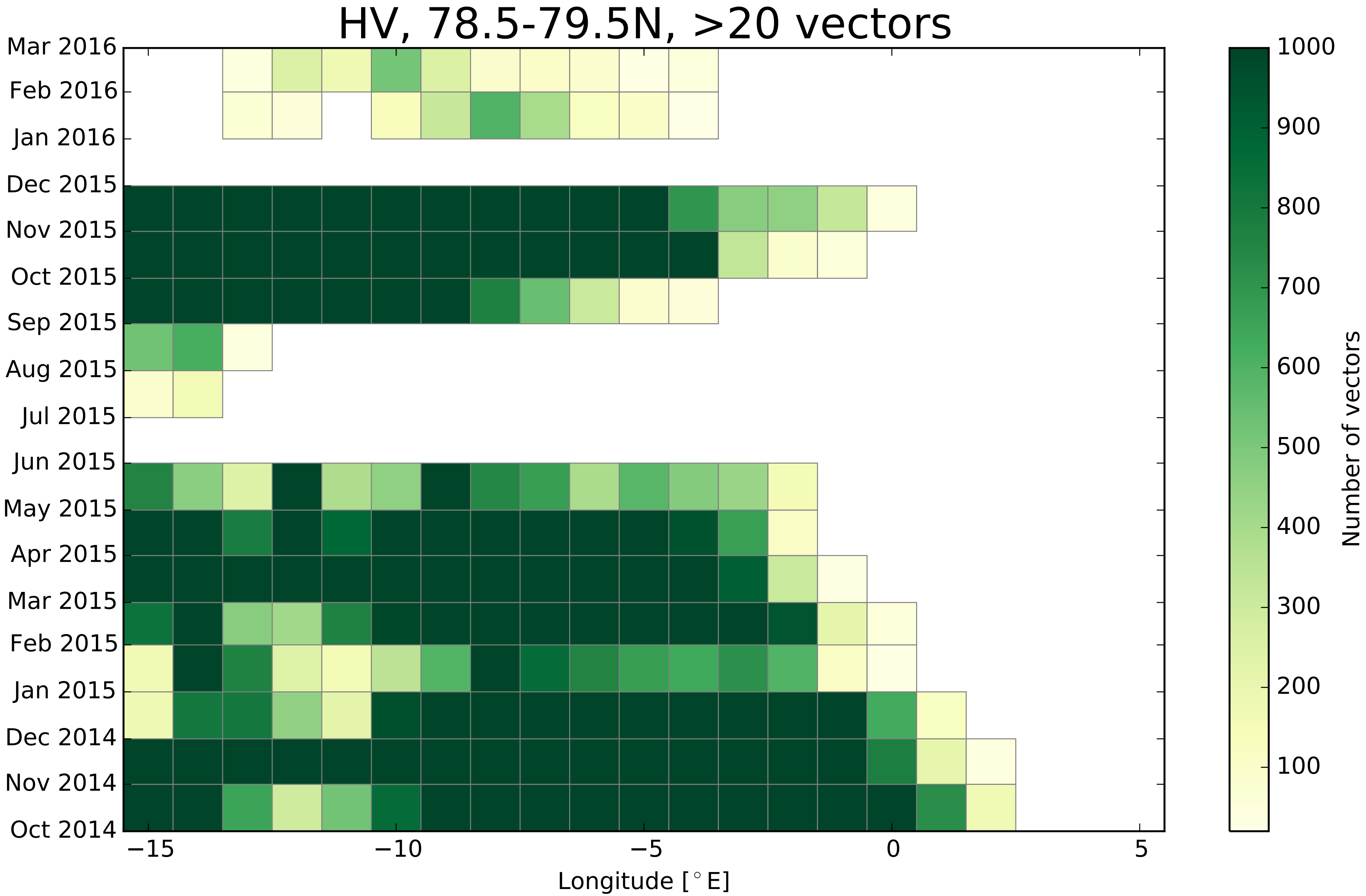

HV, 78.5-79.5N, >20 vectors
Mar 2016
Feb 2016
Jan 2016
Dec 2015
Nov 2015
Oct 2015
Sep 2015
Aug 2015
Jul 2015
Jun 2015
May 2015
Apr 2015
Mar 2015
Feb 2015
Jan 2015
Dec 2014
Nov 2014
Oct 2014
−15
−10
−5
0
5
Longitude [°E]
1000
900
800
700
600
500
400
300
200
100
Number of vectors
